# Manganese and Parkinson’s Disease: A Critical Review and New Findings

**DOI:** 10.1289/ehp.0901748

**Published:** 2010-04-19

**Authors:** Tomás R. Guilarte

**Affiliations:** Neurotoxicology and Molecular Imaging Laboratory, Department of Environmental Health Sciences, Johns Hopkins Bloomberg School of Public Health, Baltimore, Maryland, USA

**Keywords:** basal ganglia, dopamine, humans, manganese, movement disorder, neuroimaging, neurotoxicity, nonhuman primates, Parkinson’s disease, striatum

## Abstract

**Background:**

Excess accumulation of manganese (Mn) in the brain results in a neurological syndrome with cognitive, psychiatric, and movement abnormalities. The highest concentrations of Mn in the brain are achieved in the basal ganglia, which may precipitate a form of parkinsonism with some clinical features that are similar and some that are different to those in Parkinson’s disease (PD). Recently, scientists have debated the possibility that Mn may have an etiological role in PD or that it may accelerate the expression of PD.

**Objective:**

The goal of this review was to examine whether chronic Mn exposure produces dopamine neuron degeneration and PD or whether it has a distinct neuropathology and clinical presentation.

**Data source:**

I reviewed available clinical, neuroimaging, and neuropathological studies in humans and nonhuman primates exposed to Mn or other human conditions that result in elevated brain Mn concentrations.

**Data extraction:**

Human and nonhuman primate literature was examined to compare clinical, neuroimaging, and neuropathological changes associated with Mn-induced parkinsonism.

**Data synthesis:**

Clinical, neuroimaging, and neuropathological evidence was used to examine whether Mn-induced parkinsonism involves degeneration of the nigrostriatal dopaminergic system as is the case in PD.

**Conclusions:**

The overwhelming evidence shows that Mn-induced parkinsonism does not involve degeneration of midbrain dopamine neurons and that l-dopa is not an effective therapy. New evidence is presented on a putative mechanism by which Mn may produce movement abnormalities. Confirmation of this hypothesis in humans is essential to make rational decisions about treatment, devise effective therapeutic strategies, and set regulatory guidelines.

Idiopathic Parkinson’s disease (PD) is a progressive neurodegenerative disorder with a slow onset, and compared with the familial forms of the disease, it is associated with advanced age (> 55 years of age). The four cardinal signs of idiopathic PD are tremor at rest, bradykinesia (hypokinesia and akinesia), rigidity, and postural instability (Jankovic 2008; Lees et al. 2009; Tolosa et al. 2006). The diagnosis of idiopathic PD is typically based on the presence of two or more of the four cardinal signs and a response to l-dopa therapy. Unilateral tremor of the hand at rest with a frequency of 4–6 Hz is the earliest and most easily recognized symptom of PD. Autopsy-based studies have shown that the percentage of patients with confirmed PD diagnosis who exhibited resting tremor ranges from 76–100% (Hughes et al. 1993; Louis et al. 1997; Rajput et al. 1991).

Bradykinesia is defined by slowness of movements or difficulty in initiating and executing movement, and it is one of the essential signs used to diagnose idiopathic PD (Lees et al. 2009). Bradykinesia appears to correlate with the degree of dopamine deficiency in the caudate and putamen (striatum) (Vingerhoets et al. 1997). Rigidity is characterized by increased resistance of the limbs and is the result of the muscles becoming tensed and contracted so that the person feels stiff and weak. Postural instability is the loss of postural reflexes, and this occurs in the late stages of the disease. Postural instability causes patients to develop a forward or backward lean that causes them to fall. As the disease progresses, walking is affected, and patients walk in quick, small steps like they are hurrying forward in order to maintain balance.

Although the clinical diagnosis of PD is based on a combination of the four cardinal motor signs, other parkinsonian disorders also express many of these signs (Tolosa et al. 2006), and a definite diagnosis of PD requires neuropathological confirmation (Gelb et al. 1999). It has been estimated that > 10% of PD cases can be diagnosed incorrectly by movement disorder specialists when clinical signs are the only basis for diagnosis (Hughes et al. 2002). Consistent with the known deficiency of dopamine in the striatum, the clinical symptoms of PD are significantly alleviated with l-dopa therapy, the precursor substrate for the synthesis of dopamine, the chemical that is decreased in PD (Savitt et al. 2006). This is important information when one examines the hypothesis that manganese (Mn)-induced parkinsonism involves nigrostriatal dopamine neuron degeneration.

Molecular imaging is a useful strategy for the diagnosis of PD and has provided extensive evidence that PD patients exhibit decreased levels of presynaptic dopamine neuron terminal markers in the striatum (Felicio et al. 2009). This is consistent with the loss of dopamine terminals as a result of degeneration of neuronal cell bodies in the substantia nigra pars compacta (SNpc) (Figure 1). Single photon emission computed tomography (SPECT) and positron emission tomography (PET) studies have shown that PD patients exhibit decreased levels of dopamine transporters (DATs) and vesicular monoamine transporter type 2 (VMAT2) and reduced activity of dopa decarboxylase as measured by the conversion of l-dopa to dopamine in the striatum using [^18^F]-fluoro-dopa PET (Felicio et al. 2009). Postsynaptic D2-dopamine receptors (D2Rs) either are not affected or are increased in the striatum of untreated PD patients (Antonini et al. 1994; Brooks et al. 1992; Rinne et al. 1990). Similar changes in these markers have been documented in a nonhuman primate model of PD (Chen et al. 2008).

A major discovery in understanding the molecular basis of PD came about when a deficit in the concentration of the neurotransmitter dopamine was discovered in the striatum of brain samples from PD patients (Ehringer and Hornykiewicz 1960; Hornykiewicz 2006). Scientists now know that a marked loss of dopamine-containing neurons in the SNpc results in decreased dopamine levels in the caudate and putamen (Savitt et al. 2006). A hallmark neuropathological feature of SNpc dopamine neurons in PD is proteinacious intraneuronal aggregations called Lewy bodies that seem to be associated with dopamine neuron degeneration (Dauer and Przedborski 2003; Wakabayashi et al. 2007).

In summary, idiopathic PD patients exhibit resting tremor, bradykinesia, rigidity, and postural instability that is the result of decreased dopamine concentrations in the caudate and putamen from the degeneration of dopamine neurons in the SNpc that project to these brain regions (Figure 1). The early clinical symptoms of PD subjects are markedly alleviated by l-dopa therapy. The loss of dopamine neuron markers in the brain of persons with PD can be visualized and quantitatively measured using state-of-the-art neuroimaging techniques such as PET and SPECT.

## Idiopathic PD and Mn-Induced Parkinsonism: The Early Writings of James Parkinson and John Couper

In 1817, the physician James Parkinson published the first description of a neurological disorder that is now recognized by his name, Parkinson’s disease (Parkinson 1817). His monograph, titled *An Essay on the Shaking Palsy*, describes five cases with shaking palsy, a term that was vaguely employed by medical writers at the time. He commented that

[T]he first symptoms perceived are, a slight sense of weakness, with a proneness to trembling in some particular part; sometimes in the head, but most commonly in one of the hands and arms. These symptoms gradually increase in the part first affected; and at an uncertain period, but seldom in less than twelve months or more, the morbid influence is felt in some other part. Thus assuming one of the hands and arms to be first attacked, the other, at this period becomes similarly affected.

Parkinson went on to say that as the disease proceeds, “walking becomes a task which cannot be performed without considerable attention.” As the malady proceeds,

The propensity to lean forward becomes invincible, and the patient is thereby forced to step on the toes and fore part of the feet, whilst the upper part of the body is thrown so far forward as to render it difficult to avoid falling on the face . . . irresistibly impelled to take much quicker and shorter steps, and thereby to adopt unwillingly a running pace.

He then described the later stages,

As the disease proceeds toward its last stage, the trunk is almost permanently bowed, the muscular power is more decidedly diminished, and the tremulous agitation becomes violent. . . His words are now scarcely intelligible,

and he is no longer able to feed himself. “The chin is now almost immoveably bent down upon the sternum” . . . with the saliva continually trickling from the mouth. “The power of articulation is lost.”

Twenty years after the essay by James Parkinson, John Couper described the first symptoms of Mn toxicity in humans (Couper 1837). The observations made in this brief essay are important in order to understand the working conditions and the symptoms of Mn intoxication in humans. A comparison of the two essays makes it clear how the two conditions are similar and how they are different. Couper wrote,

In the chemical works of Charles Tennant and Co., a considerable number of men are employed in grinding the black oxide of manganese, to be employed in the manufacture of bleaching powder. The surface of their bodies is of course constantly covered with the manganese; the air which they breathe is loaded with it in the form of fine powder, and they are ever exposed, from neglect of cleanliness, to swallow portions of it along with their food.

It is clear from this description that working conditions were very hazardous and personal hygiene was poor. As a result, these workers were exposed to extremely high concentrations of Mn on a daily basis.

In the description of the workers who were affected by Mn poisoning, Couper went on to say that,

The loss of power is most apparent in the lower extremities, which are so considerably affected that the patient staggers, and inclines to run forward when he attempts to walk. The arms are also weakened, but only to a small extent. The patient complains that in speaking he cannot make himself heard by persons at a moderate distance, as formerly; and the inability seems to depend, not on any defect of articulation, but on weakness of voice. There is no deficiency of sensibility in any part of the body; the intellect and external senses are unimpaired; but there is an obvious expression of vacancy in the countenance, apparently from the paralyzed state of the muscles of the face. From the same cause the saliva is apt to escape from the mouth, especially during speaking. There is no tremor in any part of the body.

A comparison of the two essays indicates that certain symptoms are similar and some are different from those in PD. The Mn-exposed workers exhibited gait disturbance, a propensity to fall, masked face, hypophonia and dysphonia, and drooling. The absence of resting tremor, a prominent and early cardinal sign of PD patients, as initially described by James Parkinson, is a distinguishing observation that is different from PD in these workers heavily exposed to Mn.

Since the initial observations by Couper, other reports in the early 1900s noted the toxic effects of Mn from occupational exposures but, compared with PD, the number of studies is rather limited. The description of these subsequent Mn cases supported the initial observations by Couper but also added additional symptoms such as micrographia, dystonia (cock gait), and action and postural tremor (Table 1; for reference to these early cases, see Lee 2000; Perl and Olanow 2007). Table 1 outlines the motor symptoms from the literature related to occupational Mn exposures and other human conditions in which Mn levels are markedly increased in the brain, and the motor symptoms in idiopathic PD. The conclusion from these reports is that, although several of the clinical signs between PD and Mn-induced parkinsonism are similar, in Mn-induced parkinsonism, unlike in PD, there is an absence of resting tremor and the lack of a response to l-dopa. Also, the progression of Mn-induced parkinsonism appears to be a gait disorder of early onset with dystonia that occurs much later in the slow progression of the movement abnormalities in PD.

## Mn-Induced Parkinsonism from Human Conditions Not Related to Occupational Exposures

Besides occupational exposures, other conditions have been shown to increase accumulation of Mn in the brain and have been valuable sources of information in understanding Mn-induced neurological dysfunction, including parkinsonism.

### Psychostimulant drug abusers: ephedron

**Figure 1:**
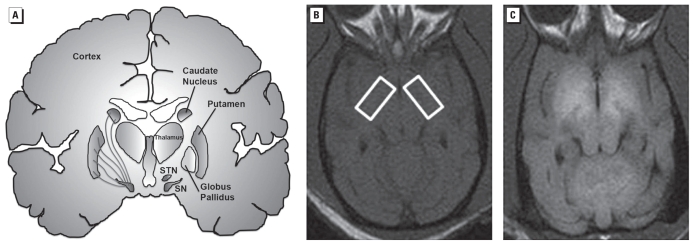
(*A*) Schematic depicting the different brain structures comprising the basal ganglia (labeled in the right hemisphere). The left hemisphere shows the nigrostriatal dopamine fibers whose cell bodies are located in the substantia nigra (SN) and innervate the caudate and putamen. These are the axonal projections that degenerate in Parksinson’s disease. T1-weighted MRI at the level of the globus pallidus of a control nonhuman primate brain (*B*; boxed areas) and a nonhuman primate brain exposed to Mn (*C*). Note the increase in signal intensity (white areas) in the Mn-exposed animal (*C*) relative to the control animal (*B*). SNT, subthalamic nucleus.

Recently, there have been a number of reports, primarily originating from Eastern European countries and Russia, of young addicts afflicted with an atypical form of parkinsonism (de Bie et al. 2007; Meral et al. 2007; Sanotsky et al. 2007; Selikhova et al. 2008; Sikk et al. 2007; Stepens et al. 2008; Varlibas et al. 2009) (Table 1). This form of parkinsonism is the result of intravenous injections of a psychostimulant drug called “ephedrone” or “Russian cocktail,” a drug in which ephedrine is oxidized using potassium permanganate and acetic acid (Sanotsky et al. 2007; Stepens et al. 2008). Typically, this homemade chemical mixture is not purified before intravenous injection, so milligram to gram amounts of Mn are injected with multiple injections occurring during the course of weeks and months. Normal Mn concentrations in whole blood are ≤ 10–12 μg/L, but the blood Mn concentrations in these young addicts have been measured at levels as high as 2,000–3,000 μg/L (Stepens et al. 2008; Varlibas et al. 2009). Consistent with the fact that high concentrations of Mn are injected, most of these individuals exhibit bilateral T1-weighted magnetic resonance imaging (MRI) hyperintensive signals in the basal ganglia and other brain regions reflecting Mn accumulation (Figure 1, Table 2).

The parkinsonian signs in these young drug-abuse subjects are consistent with those in occupationally exposed Mn workers (Table 1). An important observation on the etiological role of Mn in the parkinsonism in these young addicts is based on the fact that movement abnormalities are observed in ephedrone users in Eastern Europe and Russia where the chemical preparation uses potassium permanganate as the oxidizing agent. However, a parkinsonian syndrome has not been observed in North America, where chromate is used as the oxidizing agent rather than potassium permanganate (Selikhova et al. 2008; Stepens et al. 2008). The most parsimonious explanation is that Mn is the causative agent in this atypical form of parkinsonism.

### Patients with liver disease

There is evidence of parkinsonism associated with chronic liver disease. Patients with advanced cirrhosis have been documented with a form of parkinsonism with clinical symptoms similar to Mn-induced parkinsonism. This finding is likely because Mn is excreted in the bile, and in persons with chronic liver disease, the excretion of Mn is markedly impaired, with subsequent accumulation in the brain. The clinical symptoms associated with idiopathic PD, chronic liver disease, ephedrone abuse, and occupational exposures to Mn are described in Table 1. Some of the symptoms are common to those in PD, but there are significant differences. In conditions in which Mn is the most likely etiological agent for the parkinsonism, there is a rapid progression of the motor symptoms and early gait and postural impairment with focal dystonia (cock gait in worst cases). Further, there is an absence of resting tremor but an expression of action or postural tremor and no consistent asymmetry (Table 1). Elevated concentrations of Mn in basal ganglia structures have been measured in liver disease patients (Klos et al. 2006) and are consistent with basal ganglia hyperintensive signals in T1-weighted MRI. Increased T1-weighted MRI hyperintensive signal is not observed in PD patients. From a clinical perspective, most persons who were occupationally exposed to Mn, users of ephedron, and patients with liver disease and parkinsonism are not responsive or are minimally responsive to l-dopa therapy, the mainstay therapy that ameliorates the early movement abnormalities in PD (Table 1). Racette et al. (2001) noted an exception to this general observation; they found that welders with movement abnormalities (presumably from the Mn in the welding fume) had an excellent response to l-dopa therapy. However, a positive response to l-dopa therapy is not typical of Mn-induced parkinsonism but is representative of PD (Table 1). Further, their study has been criticized by several investigators (Ravina et al. 2001; Sadak and Schulz 2001).

## Neuroimaging Studies in Idiopathic PD and Mn-Induced Parkinsonism

With the advent of molecular imaging techniques in the 1980s, a number of neuroimaging modalities have been used to understand the structural, cellular, and molecular changes that occur in neurological and neurodegenerative diseases. In PD and conditions associated with Mn-induced parkinsonism, three principal neuroimaging modalities have been used: T1-weighted MRI, SPECT, and PET. Structural MRI and magnetic resonance spectroscopy have also been used, but to a lesser extent, and are not discussed in this review.

In human conditions where Mn is the most likely etiological agent for the parkinsonism, a high percentage of the subjects exhibited a hyperintensive signal in T1-weighted MRI that is typically first observed in the globus pallidus and in other basal ganglia structures such as the substantia nigra, caudate, and putamen (Figure 1, Table 2). This finding is because Mn is a paramagnetic metal that decreases the relaxation time in a T1-weighted MRI, which makes the signal hyperintensive. It should be noted that the basal ganglia T1-weighted MRI was normal in a small number of persons who had been occupationally exposed to Mn, who had injected the ephedron preparation, or patients who had liver disease. This obsesrvation is most likely due to the fact that the T1-weighted MRI study was performed after a significant amount of time had passed from the time of exposure to the point when the Mn had been eliminated from the brain. Importantly, for patients with liver disease, the hyperintensive T1-weighted MRI signal in the basal ganglia is normalized after liver transplantation (Aggarwal et al. 2006) that corrects the impairment in Mn excretion.

SPECT and PET studies have been used to assess nigrostriatal dopamine neuron terminal markers both in PD and in Mn-induced parkinsonism (Table 2). In PD patients, SPECT and PET studies have shown a progressive loss of DAT and VMAT2 and reduced dopa decarboxylase activity using [^18^F]-fluoro-dopa PET (Table 2). Importantly, PD patients have normal or increased levels of D2R in the striatum (Table 2). A much more limited number of SPECT or PET studies are available for conditions associated with Mn-induced parkinsonism. The most extensive series of PET and SPECT human studies that examined presynaptic and postsynaptic dopamine terminal markers was performed over several years in four workers who had been occupationally exposed to Mn in Taiwan. These workers exhibited movement abnormalities that were consistent with Mn-induced parkinsonism, and the findings from several clinical and neuroimaging studies have been recently summarized by Huang (2007). In general, these occupationally exposed workers with Mn-induced movement abnormalities had normal [^18^F]-fluoro-dopa PET, normal DAT SPECT, and a small decrease in D2R PET. A decrease in D2R levels measured by PET also has been noted in another case report (Kessler et al. 2003) (Table 2). The findings summarized by Huang (2007) are consistent with a normal synthesis of dopamine and normal levels of DAT, both of which are significantly decreased in PD patients (Table 2). Further, in Mn-induced parkinsonism, Huang (2007) observed small but significantly decreased D2R signals, which is the opposite of what is observed in PD. These findings have provided evidence for a lack of nigrostriatal dopamine neuron degeneration in workers occupationally exposed to Mn who exhibit parkinsonism.

For the most part, available evidence from SPECT and PET studies of dopamine neuron terminal markers in ephedron addicts and in liver disease patients have confirmed the lack of an effect of elevated brain Mn on dopa decarboxylase activity using [^18^F]-l-dopa PET and on DAT levels in the striatum (Colosimo and Guidi 2009; de Bie et al. 2007; Kim et al. 2007; Selikhova et al. 2008; see Table 2). In one study, Kim et al. (2002) observed significant reductions in DAT levels measured by SPECT in two workers who had been occupationally exposed to Mn (Table 2). However, both subjects exhibited an excellent response to l-dopa therapy, which is typically not observed in Mn-induced parkinsonism (Table 1). Therefore, the possibility that these two subjects were PD cases with concurrent Mn exposure is high. Also, scientists generally assumed that decreased levels of DAT as measured by PET or SPECT are representative of dopamine terminal loss in the striatum. Although this is clearly the case in PD patients where the neuropathology is well documented, this cannot be assumed in Mn-exposed subjects because studies have shown that Mn directly interacts with DAT. Chen et al. (2006) have shown that an acute dose of Mn produces a transient increase in DAT levels in the nonhuman primate striatum. This transient increase is most likely due to acute inhibition of DAT, which produces an up-regulation of the protein. Consistent with this hypothesis, a direct inhibitory effect of Mn on radioligand binding to DAT was demonstrated in the same study. That is, Mn inhibited [^3^H]-WIN 35,428 binding to DAT in neuronal membranes from rat striatum. Thus, in the study by Kim et al. (2002) and in other SPECT and PET DAT imaging studies, it is possible that the decrease in DAT measured by SPECT may not be representative of DAT loss in the striatum (or, by inference, of dopamine terminal degeneration); rather, the decrease in DAT may reflect the ability of Mn to interfere with radioligand binding to DAT. For both of the Mn-exposed cases in the Kim et al. (2002) study, there had been a long history of Mn exposure. Thus, it is possible that a progressive accumulation of Mn occurred in the striatum that reached concentrations sufficient to interfere with radioligand binding to DAT. This possibility can be explored in future studies because other dopamine terminal markers such as VMAT2 can be imaged and are less likely to be influenced by elevated levels of Mn in the striatum.

In two studies, Racette et al. described reduced [^18^F]-fluoro-dopa PET signals in two welders (2001) and in a patient with liver disease (2005). These findings suggest the possibility of nigrostriatal dopamine neuron degeneration. However, other factors need to be considered. First, in the 2001 study, 15 career welders were studied, and [^18^F]-fluoro-dopa PET was performed in only two of them. Second, all of the welders were responsive to l-dopa therapy, and this response is not consistent with Mn-induced parkinsonism. Third, none of the welders tested with T1-weighted MRI scans (8 of 15) exhibited a hyperintensive signal in the basal ganglia despite being career welders. Further, one of the two welders that had reduced [^18^F]-fluoro-dopa uptake in the posterior putamen had a positive family history of PD, which suggests the possibility of a familial form of the disease. Taking all of the information together, the most parsimonious explanation is that the parkinsonism in these welders was not likely associated with the Mn in the welding fumes. The second study (Racette et al. 2005) was a case report of a patient with alcoholic cirrhosis. This single subject had parkinsonian symptoms and elevated Mn in the blood and was responsive to l-dopa therapy, a clinical response that has not been observed in Mn-induced parkinsonism. This case had a reduction in [^18^F]-fluoro-dopa uptake throughout the caudate and putamen; however, the caudate and posterior putamen ratio was more similar to the control subjects than to the PD controls.

In summary, the evidence from neuroimaging studies have indicated that in different human conditions where Mn concentrations markedly increase in the brain to produce movement abnormalities, there is a lack of degeneration of the nigrostriatal dopaminergic terminals.

## Neurochemical and Neuropathological Studies in PD and Mn-Induced Parkinsonism

As pointed out by Perl and Olanow (2007), a very limited number of autopsy studies have been performed on workers who were exposed to Mn or on persons with other related conditions in which Mn was increased in the brain (Table 3). Most of the neuropathological studies associated with occupational Mn exposures were performed in the early 1900s and lacked detailed examination of brain tissue. Neurochemical methods used in the early studies did not have the sensitivity and specificity available today. Nevertheless, most studies described marked changes in the globus pallidus of Mn-exposed individuals, with no remarkable effects on pigmented cells of the substantia nigra, which indicates a lack of an effect on dopamine cell bodies, the only midbrain neurons that contain melanin (Table 3). Neurochemical studies of the brains of patients with liver disease have confirmed for the most part the results from neuroimaging studies: a significant decrease in D2R levels in the basal ganglia and no observed decrease in dopamine levels (Table 1). In one study, Mousseau et al. (1993) used *in vitro* autoradiography and found decreased D2R levels in the globus pallidus and putamen with no change in D1-dopamine receptors (D1Rs) in the basal ganglia of patients with liver disease (Table 3).

In general, the available neurochemical and neuropathological evidence from subjects with increased Mn concentrations in the brain suggests primary involvement of the globus pallidus, expressing the loss or shrinkage of neurons and glial cell activation. Consistent evidence has shown that D2R levels are decreased, and one study indicated no change in D1R. Most studies have indicated a lack of degeneration of midbrain dopamine neurons, which is consistent with the majority of the neuroimaging studies. Parenthetically, in the studies that indicated a loss or a shrinking of neurons in the globus pallidus or striatum of Mn-exposed subjects, no attempt has been made to identify the neuronal phenotype affected.

## Behavioral, Neuroimaging, Neurochemical, and Neuropathological Evidence of Mn Neurotoxicity in Nonhuman Primates

### Behavior

Experimental animals have been used to advance the understanding of Mn neurotoxicity. Rodent studies are not described in this review because, unlike nonhuman primates, they lack behavioral similarities to humans and are less sensitive to Mn than are humans and nonhuman primates. Nonhuman primates have improved our understanding of the effects of Mn on motor function, *in vivo* brain chemistry, and neuropathology (Table 4). Historically, nonhuman primate studies can be divided into two categories. The studies from the 1960s to the early 1990s used Mn doses that were considerably higher than those used in studies from 1995 to 2008 (Table 4). Most studies before 1995 used cumulative doses > 300 mg Mn/kg body weight. These early studies did not use neuroimaging techniques because of their more recent availability, with the exception of Newland and Weiss (1992) (Table 4), who administered very low doses of Mn and used T1-weighted MRI. Another important difference between the early studies and more recent ones has been the use of quantitative, advanced histological, neurochemical, and neuropathological techniques to analyze brain tissue. For example, some of the early studies used gross anatomical examination of brain tissue, and the analysis of neurotransmitter levels used colorimetric reactions that were prone to interference and lacked sensitivity compared with current state-of-the-art techniques (Table 4).

From a behavioral perspective, early nonhuman primate studies examined the effects of Mn on movement and coordination. It is clear from these studies that nonhuman primates exhibited movement abnormalities similar to those in Mn-exposed humans (compare Tables 1 and 4), including loss of postural stability, excitability, hypoactivity, falling, muscular rigidity, tremors of intension or action tremor, unsteady gait, loss of power in limbs, and clumsy foot movement (Table 4). From 1995, studies used cumulative Mn concentrations < 300 mg Mn/kg body weight and found more subtle movement abnormalities, including hypoactivity, deficits in fine motor control, action tremor, and, more recently, deficits in working memory (Table 4). Consistent with the human literature, the Mn-induced motor abnormalities in nonhuman primates were not responsive to l-dopa therapy (Shinotoh et al. 1995).

### Neurochemistry

The early nonhuman primate studies of Mn exposure described decreased levels of dopamine in the caudate (Neff et al. 1969), striatum and midbrain (Chandra et al. 1979), caudate and globus pallidus but not in the putamen or substantia nigra (Bird et al. 1984), and putamen but no change in the caudate (Eriksson et al. 1987). Some relevant comments are important to put these results in perspective. First, in the study by Neff et al. (1969), the monkeys in group A were injected with single doses of 200 mg Mn oxide on two different occasions. The report indicated that “2 weeks following the first injection, 1 control and 4 MnO_2_ treated monkeys died.” This was a highly unusual event and makes one question the health status of these animals prior and during treatment. They reported a significant decrease of dopamine in the caudate of group A monkeys, and a less severe dopamine deficit but still a significant one in another group of Mn-exposed animals that received a third injection (group B).

In another study, Bird et al. (1984) exposed monkeys, via inhalation, to a 30 mg/m^3^ concentration of Mn for 5 days/week, 6 hr/day, for 2 years. They found decreased levels of dopamine in the caudate and globus pallidus but not in the putamen and substantia nigra. Chandra et al. (1979) exposed male rhesus monkeys orally (MnCl_2_ at 20 mg/kg/day) for 18 months and reported significant reductions in dopamine concentrations in the striatum (−42%) and the midbrain (−15%). In this study, two observations were important. First, the brain was dissected, and the different regions were frozen at −20°C. For dopamine analysis, this temperature is not the most appropriate for storing fresh tissue, because dopamine autooxidizes readily. Second, the dopamine recovery for the method used was less than desirable and ranged from 72–84%. Although a comparison of control and treated dopamine tissue content is still possible, often in these early studies, the control and Mn-treated animals were euthanized at different times; thus potential differences in dopamine tissue content could result from differences in storage time and conditions. For example, in the study by Neff et al. (1969), Mn-exposed animals that received a higher cumulative Mn dose exhibited less dopamine loss than did animals with a lower dose whose tissues were stored for a longer time.

Eriksson et al. (1987) measured reduced concentrations of dopamine in the putamen and globus pallidus but not in the caudate, although one of the three animals analyzed did express a decrease in dopamine content in the caudate. An observation in the Eriksson et al. (1987) study that has relevance to a mechanism of action of Mn is that in the brains of these monkeys, in which they measured significant decrements in tissue dopamine concentration, they found no significant effect on glutathione concentrations in two of the three monkeys examined. In general, these early studies demonstrate differences in basal ganglia regions where dopamine concentrations appear to be decreased and the regional pattern does not follow the well-characterized loss of dopamine in idiopathic PD (Dauer and Przedborski 2003; Felicio et al. 2009).

The most consistent observation of these early studies was that Mn produced morphological changes in the globus pallidus, subthalamic nuclei, and substantia nigra pars reticulata, whereas the SNpc remained intact (Table 4). More recent studies (after 1995) that have used cumulative Mn doses < 300 mg/kg and state-of-the-art high-performance liquid chromatography (HPLC) with electrochemical detection analytical methods have provided no evidence of changes in dopamine concentrations in the caudate and putamen (Guilarte et al. 2006a, 2008; Olanow et al. 1996; Struve et al. 2007) (Table 4).

### In vitro autoradiography

*In vitro* quantitative receptor autoradiography has been used to examine dopamine neuron markers in the brain of Mn-exposed nonhuman primates (Table 4). The groups headed by Eriksson in Sweden (1992a) and by Guilarte in the United States (2006a, 2008), have used this approach extensively because not only is it quantitative, it also offers exquisite anatomical information with high resolution. Eriksson et al. (1992a) performed receptor autoradiography studies in the basal ganglia of monkeys exposed to 0.1 g Mn/month for 26 months. They indicated that this dosing regimen was comparable with what workers might inhale in dusty environments. They found that the binding of [^3^H]-mazindol to DAT (the presumed target, but see below) was reduced by 75% in the caudate and putamen of Mn-exposed animals. However, there were technical problems that need to be discussed: *a*) The authors stated that the level of nonspecific [^3^H]-mazindol binding in the caudate and putamen was 50% and 60%, respectively. This level of nonspecific binding was very high, so the specific binding signal-to-noise (nonspecific binding) ratio was very low. Typically in this type of assay, one wants a low level of nonspecific binding, in the range of 10–15% of total binding. *b*) [^3^H]-Mazindol is known to bind to other monoaminergic uptake sites besides DAT (May et al. 1994). To ensure that one is selectively measuring DAT, an antagonist for the other monoaminergic uptake sites should be included in the assay. This procedure was not performed by Eriksson et al. (1992a); thus, the effect of Mn on the [^3^H]-mazindol autoradiography results were a combination of DAT and other monoaminergic uptake sites. *c*) As pointed out, even if [^3^H]-mazindol was made selective for DAT by including antagonists for other monoaminergic transporters, a reduction in [^3^H]-mazindol binding to DAT would not necessarily reflect dopamine terminal loss, but it could represent a competitive inhibition of Mn with radioligand binding to DAT as shown by Chen et al. (2006). Parenthetically, Eriksson et al. (1992a) also found a significant decrease in [^3^H]-SCH 23390 binding to D1R in the caudate and putamen with no change in [^3^H]-spiperone binding to D2R in the same regions. These results were opposite to what Eriksson et al. (1992b) and Guilarte et al. (2008) reported with *in vivo* PET imaging and *in vitro* autoradiography—that is, decreased D2R and no effect on D1R.

Guilarte et al. (2006a, 2008) have examined the largest cohort of Mn-exposed nonhuman primates using *in vivo* and *in vitro* methods (see Table 4). In a preliminary report in which four Mn-exposed animal were used, they found no significant effect of Mn exposure (mean cumulative Mn dose, 165.5 mg Mn/kg body weight; range, 152–174 mg Mn/kg body weight) on DAT or D2R receptor levels using autoradiography or tyrosine hydroxylase immunohistochemistry, or on the concentration of dopamine and its metabolite homovanillic acid (HVA) measured by HPLC with electrochemical detection in the caudate or putamen, relative to naive controls (Guilarte et al. 2006a). These findings clearly indicated the lack of an effect of this cumulative Mn dose on the nigrostriatal dopaminergic system (but see “Neuroimaging studies,” below). In a subsequent publication with a larger number of Mn-exposed animals, Guilarte et al. (2008) essentially found the same results. Further, Mn exposure had no effect on D1R or cannabinoid receptor 1 levels in the basal ganglia. This latter study not only included a naive control group in which the animals did not receive Mn or neuroimaging studies, but it also included an imaged control group in which the neuroimaging studies were done without Mn exposure. The inclusion of an imaged control group was essential because one of the longitudinal PET studies included the administration of amphetamine (Guilarte et al. 2006a, 2008), a psychostimulant that depletes dopamine levels and down-regulates DAT. This control group provided valuable information for assessing the effect of amphetamine alone. Although several of the dopamine neuron markers that were analyzed postmortem, such as dopamine and DOPAC (3,4-dihydroxyphenylacetic acid) in the putamen, DAT and VMAT2 in the caudate and putamen and D2R in the putamen were significantly lower in Mn-exposed animals than in naive controls; they were not different from all controls when the imaged control group was included. The values for these parameters in the imaged control animals were actually lower than those for the Mn-exposed animals. The fact that studies of Mn-exposed animals that received amphetamine with PET had higher levels of these dopamine markers than did imaged controls that received the same amphetamine with PET but no Mn suggests that Mn has an “antagonistic effect” on the action of amphetamine on these dopaminergic markers. Because this action on dopaminergic markers is mediated by DAT, these results provide evidence that Mn interacts directly with DAT and are consistent with other studies that indicate an interaction of Mn with DAT (Anderson et al. 2007; Chen et al. 2006; Ingersoll et al. 1999).

### Neuroimaging studies: MRI and PET

Similar to the studies involving humans exposed to Mn, several investigators have performed neuroimaging studies of nonhuman primates to examine the distribution of Mn in the brain by T1-weighted MRI and brain chemistry changes using PET. The T1-weighted MRI studies (Newland et al. 1989; Newland and Weiss 1992) (Table 4) showed that Mn first accumulates in the globus pallidus and produces a hyperintensive signal in T1-weighted MRI even at relatively low doses of Mn. Thus, it is a sensitive method for detecting small increases in brain Mn concentrations. These findings have been confirmed by other investigators, and subsequent studies have shown that although the basal ganglia is one of the first to accumulate Mn, other brain structures also accumulate the metal, albeit to a lesser extent (Dorman et al. 2006; Ingersoll et al. 2006b).

PET imaging in nonhuman primates exposed to Mn was first performed by Eriksson (1992b) (Table 4). They studied two monkeys exposed to Mn for 16 months that received [^11^C]-nomifensine PET to measure DAT (although this ligand also recognizes other monoaminergic transporters), [^11^C]-raclopride PET for D2R, and [^11^C]-l-dopa for l-dopa decarboxylase activity. They found a progressive decrease in [^11^C]-nomifensine binding as a function of cumulative dose in both animals. One of the animals had a transient decrease in [^11^C]-raclopride binding to D2R that returned to baseline by the end of the exposure period, and no effect of Mn treatment on [^11^C]-l-dopa PET was observed. These studies indicate that dopamine neurons are not degenerating, but there is a potential loss of dopamine terminals based on the [^11^C]-nomifensine PET. However, a firm conclusion that chronic Mn results in the loss of DAT and presumably dopamine neuron terminals in the striatum cannot be made because [^11^C]-nomifensine is not a selective DAT ligand and because Mn can directly inhibit ligand binding to DAT.

Shinotoh et al. (1995) performed PET studies in three monkeys (not all monkeys received the three different types of PET studies) that were chronically treated with Mn. These investigators found no significant effect of Mn on [^18^F]-fluoro-l-dopa PET, [^11^C]-raclopride PET, or [^18^F]-deoxyglucose PET despite the fact that the animals exhibited hypoactivity (Table 4). Thus, their work is also consistent with the lack of degeneration of nigrostriatal dopaminergic neurons.

### Neuroimaging studies: new PET findings

Since the study by Shinotoh et al. (1995), there has been a lack of neuroimaging studies to examine the effects of chronic Mn exposure on brain chemistry in nonhuman primates. Guilarte et al. (2006a, 2006b, 2008) have recently undertaken a large multidisciplinary effort to more broadly examine the longitudinal effects of chronic Mn exposure on behavior, *in vivo* brain chemistry, and brain pathology in the same animals (only the PET studies related to the dopaminergic system were described here). They performed PET studies of presynaptic and postsynaptic “structural proteins” such as DAT ([^11^C]-methylphenidate as the PET radioligand) and D2R ([^11^C]-raclopride as the PET radioligand) to examine various markers of the dopaminergic synapse. A PET study to assess dopaminergic function was also included. Although [^18^F]-fluoro-dopa PET is a perfectly valid and useful functional marker of dopaminergic activity, this method interrogates the ability of dopamine neurons to synthesize dopamine, and it may indirectly measure the dopamine-releasing capacity of dopamine neurons. Guilarte et al. (2006a, 2008) used a more direct method to assess *in vivo* dopamine release using a bolus plus continuous infusion of [^11^C]-raclopride (D2R ligand) with amphetamine challenge (Zhou et al. 2006). This type of PET study is based on the ability of endogenous dopamine (released by the administration of amphetamine) to compete with the binding of [^11^C]-raclopride to D2R (Laruelle 2000). An animal with a high capacity to release dopamine will produce a large decrease in the amount of [^11^C]-raclopride uptake in the striatum, whereas an animal with a low capacity will produce a smaller change or no change. These studies showed that the most significant effect of chronic Mn at all of the cumulative doses of Mn administered was a marked inhibition (> 50% on average) of *in vivo* dopamine release in the striatum measured by PET (Guilarte et al. 2006a, 2008) (Table 4). The effect of Mn on *in vivo* dopamine release was observed in the absence of a change in DAT levels and with a small but significant decrease on D2R levels in the striatum (Guilarte et al. 2008). Therefore, consistent with human and other nonhuman primate studies, this work also showed a lack of dopamine neuron degeneration. However, these studies provided new information: although chronic Mn exposure did not result in dopamine neuron degeneration, dopamine neurons were dysfunctional because they had a reduced capacity to release dopamine. Neurochemical studies in the brain of the same animals that received PET studies showed that the marked effect of Mn on *in vivo* dopamine release measured by PET was not due to a reduction in tissue dopamine levels, because dopamine concentrations were not significantly different from all controls (Guilarte et al. 2006a, 2008). It appears that the deficit of *in vivo* dopamine release is associated with the ability of Mn to disrupt presynaptic release mechanisms.

## Conclusions

The available evidence from human and nonhuman primate studies using behavioral, neuroimaging, neurochemical, and neuropathological end points provides strong support to the hypothesis that, although excess levels of Mn accumulation in the brain results in an atypical form of parkinsonism, this clinical outcome is not associated with the degeneration of nigrostriatal dopaminergic neurons as is the case in PD. In fact, the new evidence suggests that it may be the dysfunction of this system or the inability to release dopamine that produces the movement abnormalities documented in Mn-exposed subjects. This new finding is consistent with the fact that l-dopa therapy does not provide a benefit to the Mn-induced movement abnormalities as it does in PD (Lu et al. 1994) (Table 1). This is because Mn-induced motor dysfunction does not seem to be a problem of decreased synthesis or concentration of dopamine in presynaptic terminals, but rather a problem of the ability to release the available dopamine. These findings provide a starting point and a new avenue of research to delineate a putative mechanism of Mn-induced movement abnormalities.

Although the latest research clearly suggests that the effects of Mn on presynaptic aspects of the dopaminergic synapse need further exploration, delineating its effects on postsynaptic proteins of the dopaminergic synapse or other neuronal systems downstream and on the role of glial cells is essential in order to achieve a greater understanding of the neurotoxicity of Mn. The continued use of neuroimaging techniques that have already been used to study Mn neurotoxicity, as well as some others such as diffusion tensor imaging, are also likely to provide new information. Ultimately, the most significant studies in understanding Mn-induced parkinsonism are those in humans. From this perspective, persons who inject high doses of Mn in the ephedron preparations and workers who are occupationally exposed to moderate levels of Mn are cohorts in which comprehensive neurological assessments along with neuroimaging studies can provide longitudinal information about Mn neurotoxicity, its reversibility, and the usefulness of therapeutic strategies. Finally, although the preponderance of the behavioral, neuroimaging, and neuropathological evidence indicates that chronic Mn exposure does not cause degeneration of nigrostriatal dopamine neurons and PD, the parkinsonism and other neurological effects resulting from chronic Mn exposure are highly debilitating, and it should not be treated with any less importance.

## Figures and Tables

**Table 1:** Clinical symptoms of parkinsonism in idiopathic PD and in human conditions with elevated brain Mn concentrations.

Reference	Category	Resting tremor	Action/postural tremor	Bradykinesia	Rigidity	Postural instability	Gait disorder	Dystonia	Micrographia	Difficulty walking backward, turning, pull-test	Hypophonia, dysphonia/dysarthria	Falls	l-Dopa response
Jankovic 2008	IPD	+		+	+	+	+	+ Secondary	+	+	+		Excellent
Lee 2000	Mn-O		+	+	+	+	+		+	+	+	+	
Yamada et al. 1986	Mn-O			+	+	+	+	+					
Cook et al. 1974	Mn-O		+	+		+	+		+	+	+		Minimal
Mena et al. 1967	Mn-O		+	+	+	+	+	+	+	+	+		
Huang 2007 (summary of Taiwan cohort)	Mn-O	−	+	+	+	+	+	+	+	+	+		Minimal to none
Kessler et al. 2003	Mn-O	+	+	+	+	+	+			+	+	+	None
Kenangil et al. 2006	Welding			+	+	+	+	+				+	None
Bowler et al. 2006	Welding		+	+	+		+	+					None
Josephs et al. 2005	Welding	−	+	+		+	+			+	+		Minimal
Racette et al. 2001	Welding	+		+	+	+							Excellent
Sadek et al. 2003	Welding		+		+	+	+	+				+	None
Meral et al. 2007	Ephedron	Some	+	+	+	+	+	+		+	+		None
Sikk et al. 2007	Ephedron					+	+	+		+	+		
de Bie et al. 2007	Ephedron			+	+	+	+		+	+	+		None
Sanotsky et al. 2007	Ephedron			+	+	+	+	+	+	+	+	+	None
Varlibas et al. 2009	Ephedron		+	+		+		+		+			Minimal to none
Stepens et al. 2008	Ephedron	−		+	+	+	+	+	+	+	+	+	None
Selikhova et al. 2008	Ephedron			+		+	+	+	+	+	+		None
Colosimo and Guidi 2009	Ephedron		+	+	+	+		+		+	+	+	None
Nagatomo et al. 1999	PN	Some			+		+	+		+	+	+	
Burkhard et al. 2003	LD	−	+	+	+	+	+	+		+	+	+	Minimal
Klos et al. 2006	LD	−	+	+	+		+						None
Klos et al. 2005	LD		+	+	+	+	+						Minimal
Faviani et al. 2007	LD		+	+	+	+	+		+		+	+	Partial
Kim et al. 2007	LD		+	+	+	+	+			+			None
Schaumberg et al. 2006	Mn-O/LD	−	+	−	−	+	+	+			+		Not tested

Abbreviations: IPD, idiopathic PD; LD, liver disease; Mn-O, occupational Mn exposure; PN, parenteral nutrition; secondary, later in time; some, minimal; +, the symptom was present; −, the symptom was not present; blank spaces, neither was indicated.

**Table 2:** Brain imaging studies in idiopathic PD and in human conditions that show elevated concentrations of Mn in the brain.

Reference	Category	T1-MRI	PET/SPECT	Blood or tissue Mn
Felicio et al. 2009	IPD	NTD	[^18^F]-fluoro-dopa PET DAT/SPECT/PET VMAT2/PET (all significantly decreased)	NTD
Brooks et al. 1992	IPD	NTD	D2R/PET (increased or normal)	NTD
Leenders 2003	IPD	NTD	D2R/PET (increased or normal)	NTD
Huang 2007 (summary of Taiwan cohort)	Mn-O	↑	[^18^F]-fluoro-dopa PET (normal) DAT/SPECT (normal) D2R/PET (small decrease)	↑
Kim et al. 2002	Mn-O	↑	DAT/SPECT (decreased)	↑
Bowler et al. 2006	Welding	↑	NP	↑
Josephs et al. 2005	Welding	↑	NP	↑
Racette et al. 2001	Welding	No signal	[^18^F]-fluoro-dopa PET (major reduction)	NP
Sadek et al. 2003	Welding	↑	NP	↑
Meral et al. 2007	Ephedron	↑	NP	NP
de Bie et al. 2007	Ephedron	↑	[^18^F]-fluoro-dopa PET (minor reduction)	↑
Sanotsky et al. 2007	Ephedron	↑	NP	NP
Varlibas et al. 2009	Ephedron	↑	NP	↑
Stepens et al. 2008	Ephedron	↑	NP	↑
Selikhova et al. 2008	Ephedron	↑	DAT/SPECT (normal)	↑
Colosimo and Guidi 2009	Ephedron	Normal	DAT/PET (normal)	NP
Racette et al. 2005	LD	↑	[^18^F]-fluoro-dopa PET (major reduction)	↑
Burkhard et al. 2003	LD	↑	NP	↑
Klos et al. 2006	LD	↑	NP	↑
Klos et al. 2005	LD	↑	NP	↑
Faviani et al. 2007	LD	↑	NP	↑
Kim et al. 2007	LD	↑	DAT/SPECT (normal)	↑
Schaumberg et al. 2006	Mn-O/LD	↑	NP	↑
Kessler et al. 2003	Mn-O	NP	D2R/PET (decreased)	NP

Abbreviations: IPD, idiopathic PD; LD, liver disease; Mn-O, occupational Mn exposure; NP, not performed; NTD, not typically done. PET/SPECT studies were done in the striatum (caudate and putamen). Arrows indicate increased T1-weighted MRI signal in the globus pallidus and other basal ganglia structures.

**Table 3:** Neurochemical studies in human postmortem brain tissue.

Reference	Category	Neurochemistry/neuropathology
Bernheimer et al. 1973	Mn-O	Putamen, pallidum, and red nucleus exhibited generalized astroglial activation Mild degree of pallidal atrophy Decreased DA levels in caudate, putamen, and SN Lewy bodies in SN
Yamada et al. 1986	Mn-O	Loss of nerve cells in the pallidum Moderate decrease in the number of large nerve cells, and shrinking of cells Pigmented cells of the SN were intact
Perl and Olanow 2007	Mn-O	Describes studies from the early 1900s to 2004 indicating pathological effects in GP with no remarkable effects in SN
Butterworth et al. 1995	Liver disease	Loss of D2R in GP
Mousseau et al. 1993	Liver disease	[^3^H]-SCH 23390 autoradiography (D1R) unchanged in all basal ganglia regions tested [^3^H]-Spiperone autoradiography (D2R) decreased in GP and putamen; no change in caudate
Bergeron et al. 1989	Liver disease	DA/HPLC not changed in the caudate; HVA increased

Abbreviations: DA, dopamine; GP, globus pallidus; Mn-O, occupational Mn exposure; SN, substantia nigra.

**Table 4:** Behavioral, neuroimaging, and neurochemical studies in Mn-exposed nonhuman primates.

Reference	Species	Route	∑Mn dose	Behavior	Neuroimaging	Neuropathology/neurochemistry
Pentschew et al. 1963	Rhesus (5 monkeys, only 1 described)	im (Mn dioxide)	695 mg Mn/kg (calculated)	Excitability Loss of postural stability Falling upon jumping Clumsiness No cogwheel, tremor, or involuntary movements	ND	Neuronal loss and gliosis in subthalamic nucleus and medial segment of GP Gliosis in SN (?) Atrophic nerve cells in lateral pallidum
Neff et al. 1969	Squirrel monkey (control = 4; Mn-group A = 5; Mn-group B = 6)	sc (Mn dioxide)	379 mg Mn/kg in highest dose group (calculated)	Muscular rigidity Impaired equilibrium Tremors on intension Impaired equilibrium	ND	Decreased DA and NE in caudate
Chandra et al. 1979	Rhesus (4)	Oral	23,580 mg Mn/kg (MnCl_2_) (calculated)	ND	ND	Decreased DA in striatum and midbrain
Bird et al. 1984	Rhesus (4)	Inhalation (Mn oxide)	30 mg Mn/m^3^ air (exposure rate)	No behavioral or neurological abnormalities	ND	Decreased DA in caudate and GP but not in putamen or SN
Eriksson et al. 1987	Rhesus (4)	sc (Mn oxide)	1,543 mg Mn/kg (calculated)	Hyperactive tending to fall then hypoactive Unsteady gait Action tremor Loss of power in limbs Clumsy hands and feet movement	ND	Severe neuronal loss and gliosis in GP, rest of brain appears normal Decreased DA and DOPAC in putamen but not changed in the caudate HVA levels normal in most animals Decreased dopa decarboxylase activity in putamen of two-thirds of the animals ChAT activity reduced in GP of all animals GAD activity unaffected Glutathione levels decreased in the striatum, GP, and SN of one third of the animals
Newland et al. 1989	Long-tailed macaque (3)	iv and inhalation (MnCl_2_)	30–50 mg Mn/kg (injection)	ND	↑ T1-MRI hyperintensity basal ganglia	ND
Newland and Weiss 1992	Cebus (3)	iv (MnCl_2_)	40–60 mg Mn/kg	Action tremor	↑ T1-MRI hyperintensity basal ganglia	ND
Eriksson et al. 1992a	Long-tailed macaque (3)	sc (Mn oxide)	333–444 mg Mn/kg (calculated)	Decreased activity in ½ of the Mn-exposed animals	ND	DAT/autorad (decreased in caudate and putamen, no change in GP) D1R/autorad (decreased in caudate and putamen, no change in GP) D2R/autorad (no change in any region) mAChR/autorad (no change in any region) GABAaR/autorad (no change in any region)
Eriksson et al. 1992b	Long-tailed macaque (2)	sc (Mn oxide)	680 mg Mn/kg (calculated)	Unsteady gait Hypoactivity Clumsiness of hands and feet	DAT/PET (60% decrease) D2R/PET (40% decrease then normalized) l-Dopa/PET (normal) ↑ T1-MRI hyperintensity in basal ganglia	ND
Shinotoh et al. 1995	Rhesus (3)	iv (MnCl_2_)	71–87 mg Mn/kg	Two animals hypoactive, one normal Not responsive to l-dopa	D2R/PET l-Dopa/PET (normal) FDG/PET (normal) ↑T1-MRI hyperintensity basal ganglia	Minimal cell loss and prominent gliosis in GP and lesser degree in SNpr (normal) SNpc relatively spared
Olanow et al. 1996	Rhesus (3) (same animals as Shinotoh et al. 1995)	iv (MnCl_2_)	71–87 mg Mn/kg	Two animals hypoactive, one normal Not responsive to l-dopa	See Shinotoh et al. (1995)	Minimal cell loss and prominent gliosis in GP and to lesser degree in SNpr SNpc appeared normal Striatal DA and HVA levels are normal in two affected animals
Dorman et al. 2006	Rhesus (20)	Inhalation	Various levels of exposure	ND	↑ T1-MRI hyperintensity in basal ganglia	ND
Chen et al. 2006	Baboon (2)	sc and iv	10–100 mg Mn/kg (acute)	ND	DAT/PET (transient increase)	ND
Guilarte et al. 2006a	Cynomolgus (3–5)	iv	165.5 ± 4.7 mg Mn/kg (range = 152–174)	Subtle deficits in fine motor control Small decrease in activity	DAR/PET (significantly decreased) DAT/PET (normal) D2R/PET (normal)	DAT/autorad (normal) D2R/autorad (normal) TH/immunohistochemistry (normal) DA-HVA/HPLC (normal)
Guilarte et al. 2006b	Cynomolgus (4)	iv	165.5 ± 4.7 mg Mn/kg (range = 152–174)	See Guilarte et al. 2006a	↑ T1-MRI hyperintensity in basal ganglia and other brain regions MRS changes in regions outside basal ganglia	See Guilarte et al. (2006a)
Struve et al. 2007	Rhesus (20)	Inhalation	Various levels of exposure	ND	ND	DA-DOPAC-HVA/HPLC (normal)
Guilarte et al. 2008	Cynomolgus (13; includes four animals from Guilarte et al. 2006a)	iv (Mn sulfate)	68–250 mg Mn/kg	See Schneider et al. 2006, 2009	DAR/PET (significantly decreased) DAT/PET (normal) D2R/PET (small decrease)	DAT/autorad (normal) D2R/autorad (normal) D1R/autorad (normal) CB1/autorad (normal) TH/immunohistochemistry (normal) DA-HVA/HPLC (normal)

Abbreviations: autorad, autoradiography; CB1, cannabinoid receptor 1; ChAT, choline acetyltransferase; DA, dopamine; DAR, dopamine release; DOPAC, 3,4-dihydroxyphenylacetic acid; D2R, dopamine receptor; FDG, fluorodeoxyglucose; GABAaR, γ-aminobutyric acid A receptor; GAD, glutamic acid decarboxylase; GP, globus pallidus; im, intramuscular; iv, intravenous; mAChR, muscarinic acetylcholine receptor; MRS, magnetic resonance spectroscopy; ND, not determined; NE, norepinephrine; sc, subcutaneous; SN, substantia nigra; SNpr, substantia nigra pars reticulata; TH, tyrosine hydroxylase. Arrows indicate increased T1-weighted MRI signal in the globus pallidus and other basal ganglia structures.
